# Thyroid Hormone T3 Induces DNA Damage Response in Breast Cancer Cells

**DOI:** 10.3390/ijms27020668

**Published:** 2026-01-09

**Authors:** Sahar Movshovitz, Liat Anabel Sinberger, Keren Trabelsi, Amit Bar-on, Amir Sonnenblick, Mali Salmon-Divon, Tamar Listovsky

**Affiliations:** 1 Department of Molecular Biology, Ariel University, Ariel 4077625, Israel; saharm@ariel.ac.il (S.M.); trabelsi.keren@gmail.com (K.T.); amitb@ariel.ac.il (A.B.-o.); malisa@ariel.ac.il (M.S.-D.); 2 Institute of Oncology, Tel Aviv Sourasky Medical Center, Tel Aviv 6423906, Israel; amirson@tlvmc.gov.il; 3 Sackler Faculty of Medicine, Tel Aviv University, Tel Aviv 6997801, Israel; 4 Adelson School of Medicine, Ariel University, Ariel 4077625, Israel

**Keywords:** thyroid hormone (TH), T3, DNA damage, breast cancer, Oncotype DX

## Abstract

Thyroid hormones (THs) regulate metabolism, proliferation, and genomic stability. Clinical studies have linked levothyroxine therapy with higher Oncotype DX Recurrence Scores in breast cancer (BC), suggesting a potential effect of thyroid hormone signaling on genomic risk. Here, we investigated the impact of triiodothyronine (T3) on DNA damage and repair pathways in estrogen receptor-positive T47D breast cancer and non-tumorigenic MCF10A cells. RNA sequencing revealed significant upregulation of RAD51 and enrichment of DNA repair pathways following 24 h T3 exposure. Consistently, T3 increased γH2AX and 53BP1 nuclear foci, indicating transient activation of the DNA damage response (DDR). These effects were transient, returning to baseline after 48 h, suggesting cellular adaptation. T3 also enhanced proliferation at 10 μM but inhibited growth at higher concentrations. Our findings indicate that acute exposure to T3 induces transient genomic stress, providing a potential mechanistic basis for the observed association between thyroid hormone therapy and increased BC recurrence risk.

## 1. Introduction

The thyroid hormone (TH) receptors (THRs) are known to play a role in regulating cellular processes such as aging, cancer, and degenerative diseases, suggesting they possess tumor suppressor activity [1,2,3]. In the clinical context, estrogen receptor-positive (ER+) and human epidermal growth factor receptor 2-negative (HER2-negative) support breast cancer (BC) treatment decisions, which are guided by the tumor’s genomic signature, such as Oncotype DX Recurrence Score (RS). Genomic risk, as determined by the RS, serves as a prognostic and predictive measure for quantifying the risk of disease recurrence [4].

Emerging clinical data suggest a link between thyroid hormone supplementation and genomic risk assessment in BC [5]. A retrospective cohort study demonstrated that the use of levothyroxine-a commonly prescribed non-oncology medication for hypothyroidism-was significantly associated with a high Oncotype DX RS, when compared to patients not receiving non-oncology drugs. This association remained significant even after normalization against traditional covariates such as tumor size, grade, and lymph node involvement, establishing levothyroxine as an independent influence on the RS [6]. This finding is clinically relevant, as it implies that levothyroxine use may influence the likelihood of recommending adjuvant chemotherapy. The overall suggestion from this prior work is that levothyroxine may influence genomic risk, though not via proliferation, as Ki67 levels were unchanged, but potentially through its impact on hormone-dependent signaling, specifically progesterone-related features.

The mechanistic link between thyroid hormone action, cellular stress, and genomic integrity provides a strong basis for connecting these clinical observations to DNA damage. One of the most prominent actions of TH is the regulation of mitochondrial function and basal metabolism. Studies have demonstrated that T3 (triiodothyronine), the active thyroid hormone, can induce DNA damage and premature senescence in cultured cells and tissues of young hyperthyroid mice, an action primarily mediated by the Thyroid Hormone Receptor β (THRB) isoform [7]. Notably, the mechanism for DNA damage induction involves several pathways. Binding to THRB initiates an ATM (ataxia telangiectasia mutated)/PRKAA (adenosine monophosphate–activated protein kinase) signal transduction cascade. This signaling axis increases the expression of mitochondrial genes, leading to a demonstrable enhancement of mitochondrial respiration and oxygen consumption. This augmented mitochondrial activity results in enhanced electron leak and the subsequent production of mitochondrial Reactive Oxygen Species (ROS), leading to cellular oxidative stress. The resulting oxidative stress can cause DNA lesions, including DNA double-strand breaks (DSBs) [8,9]. This is confirmed by the increase in the oxidative biomarker (8-OH-dG) in nuclear foci, which strongly colocalizes with TP53BP1 (a marker of DSBs). This damage triggers an activated DNA damage response (DDR), which, if persistent, leads to premature senescence. The DNA damage has been shown to be oxidative in nature, as treatment with the antioxidant N-acetyl-l-cysteine (NAC) prevents the formation of the DSB foci.

This mechanism suggests that chronic hyperthyroidism or inappropriate T3 therapy could potentially contribute to genomic instability. Further supporting this concept, studies on peripheral blood mononuclear cells (PBMCs) have shown that exposure to T3 elevates DNA migration (a measure of primary DNA damage) in both normal cells and, notably, in those from obese, prediabetic, and type 2 diabetes mellitus (T2DM) patients, suggesting increased sensitivity to T3’s genotoxic effects across metabolic risk groups [10].

Given the prior correlation between levothyroxine usage and elevated genomic risk in BC patients, and the established mechanism linking T3 to oxidative DNA damage, the present study investigates the effects of T3 supplementation on gene expression. We used breast cancer cell line T47D, along with the non-tumorigenic, immortalized mammary epithelial cell line MCF10A, to assess T3 effect on gene expression. RNA-seq enrichment suggests DDR pathway activation, and we observed an induction in Rad51 and 53BP1. This work provides ground for epidemiological observations of women treated with T3, which may require a more rigid surveillance scheme to identify potential cancer recurrence events.

## 2. Results

### 2.1. T3 Supplementation Modulates Cell Proliferation

To investigate the effect of triiodothyronine (T3) on cell proliferation, we utilized the breast cancer cell line T47D, which is estrogen receptor-positive (ER+), progesterone receptor-positive (PR+), HER2-negative luminal B subtype, and the non-tumorigenic, immortalized mammary epithelial cell line MCF10A. Cells were first cultured in estrogen-depleted medium for 24 h to eliminate endogenous hormonal influences. Subsequently, they were treated with T3 at concentrations of 0, 1 μM, 10 μM, and 100 μM for 24 or 48 h.

**Figure 1:**
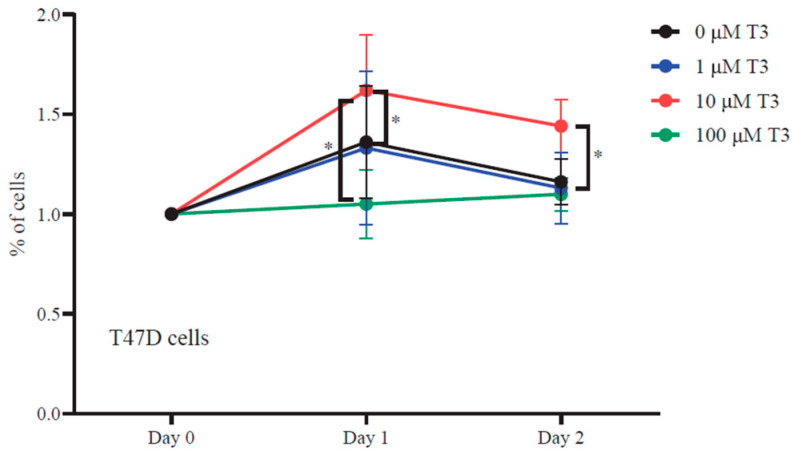
T3 supplementation modulates T47D proliferation. Cells were treated as indicated, and XTT proliferation assay was performed. A 24 h supplement of 10 μM T3 induced cell growth while 100 μM prevented cell proliferation. The average of three independent biological experiments that were performed in triplicate was plotted. *p*-value was determined using a two-way ANOVA test, SD = 1. (*: *p* ≤ 0.05).

Our results demonstrate that both T47D and MCF10A cells maintained proliferative capacity in the absence of estrogenic hormones (Figure 1). Notably, treatment with 10 μM T3 for 24 h significantly enhanced cell proliferation in both cell lines compared to untreated controls. In contrast, exposure to high-dose T3 (100 μM) resulted in growth inhibition, whereas treatment with low-dose T3 (1 μM) yielded proliferation rates comparable to untreated cells. The apparent reduction in cell number observed at 48 h across all conditions is attributed to overconfluence-induced cell death rather than a direct effect of T3 treatment.

### 2.2. T3 Induced Expression of DNA Damage Genes

**Figure 2:**
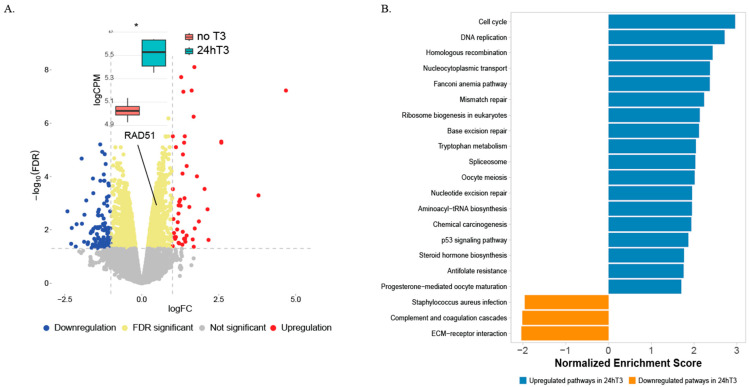
RNA expression profile of T47D cells. (**A**) Volcano plot of differentially expressed genes (DEGs) between 24hT3 and no T3 cell lines, showing log fold change (logFC) on the *x*-axis and −log10(FDR) on the *y*-axis. Point color indicates upregulated (red), downregulated (blue) or FDR significant (yellow) genes. boxplot representing the expression levels of RAD51 gene in 24hT3 and no T3 cell lines. For each treatment *n* = 3 biological repeats. (**B**) Gene Set Enrichment Analysis (GSEA) of DEGs comparing 24hT3 and no T3 cell lines. (*: *p* ≤ 0.05).

To assess the impact of T3 on gene expression, T47D cells were treated with 10 μM T3 for 24 h, after 24 h of T3 depletion, and transcriptomic analysis was performed. Differential gene expression analysis revealed 49 upregulated and 117 downregulated genes (FDR < 0.05), including significant upregulation of RAD51, a key mediator of homologous recombination repair (Volcano plot, Figure 2A). Gene Set Enrichment Analysis (GSEA) further identified activation of multiple DNA repair pathways, including homologous recombination, mismatch repair, base excision repair (BER), nucleotide excision repair (NER), and non-homologous end joining (NHEJ) (Figure 2B, GSEA KEGG T47D).

### 2.3. T3 Induces Expression of DNA Damage Response

**Figure 3:**
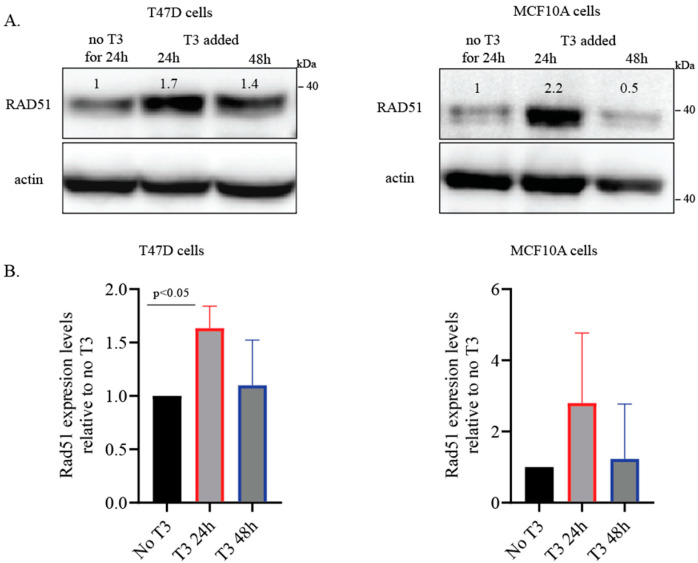
T3 supplementation increases expression of DNA damage proteins. (**A**) T47D and MCF10A cell lines present an induction in RAD51 protein levels after 24 h treatment with 10 μM of T3, and after 48 h, RAD51 levels return to near-basal levels. (**B**) Quantification of RAD1 expression levels, relative to 24 h T3 depletion. All blots that contributed to the quantification are presented in Figure S1. For all, *n* = 3 independent experiments. *p*-value was calculated using a one-tailed *t*-test based on the directional hypothesis derived from the RNA-seq data, SD = 1.

To validate the transcriptomic findings, we assessed RAD51 protein levels in T47D and MCF10A cells. After 24 h of T3 depletion, T3 (10 μM) was added for 24 h. In both cell lines, RAD51 expression increased approximately by two-fold after 24 h exposure to T3. By 48 h, RAD51 levels had returned to baseline, indicating cellular adaptation to T3 treatment (Figure 3A). These changes were more pronounced in MCF10A cells, where levels changed dramatically, whereas the T47D cells presented moderate elevation in RAD51 levels; however, these changes were more consistent and significant (Figure 3B).

**Figure 4:**
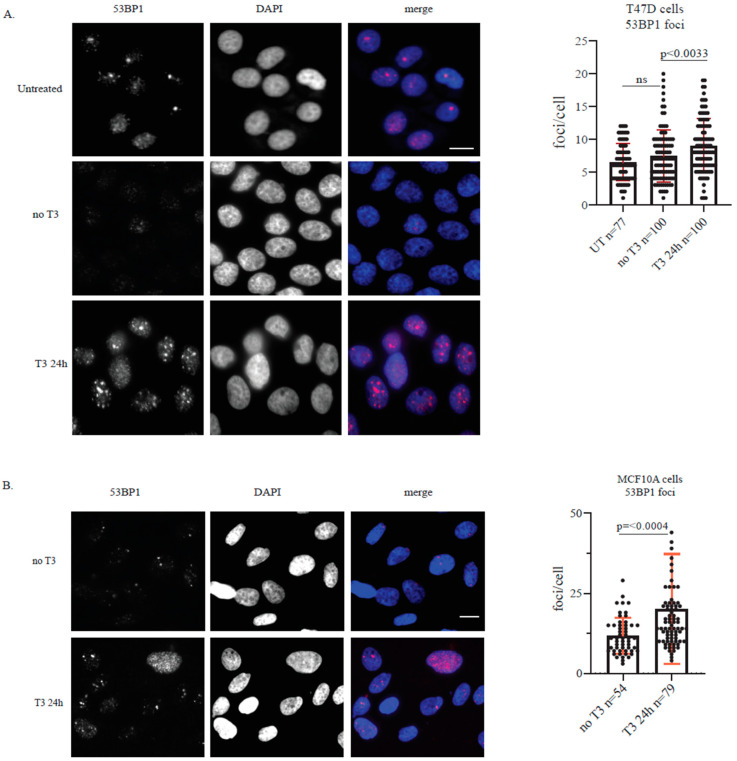
T3 supplementation induces expression of 53BP1. (**A**) T47D cell line presents an induction in 53BP1 foci after 24 h treatment with 10 μM of T3. Representative images of 53BP1 foci (**left panel**). 53BP1 foci quantification (**right panel**). (**B**) MACF10A cell line presents an induction in 53BP1 foci after 24 h treatment with 10 μM of T3. Representative images of 53BP1 foci (**left panel**). 53BP1 foci quantification (**right panel**). For all, bar 0.5 μm, *p*-value was calculated using a two-tailed *t*-test, SD = 1.

Following confirmation of RAD51 upregulation-a key protein in the homologous recombination (HR) pathway-we investigated the effect of T3 on 53BP1, a central protein in the non-homologous end joining (NHEJ) pathway. Immunofluorescence staining for 53BP1 revealed a two-fold increase in the number of nuclear foci in both T47D (Figure 4A) and MCF10A cells after 24 h treatment with T3 (10 μM), compared to T3-depleted controls (Figure 4B). No such increase was observed in cells maintained without T3, further supporting a T3-specific effect.

The concurrent increase in RAD51 and 53BP1 protein levels following short-term T3 exposure suggests that T3 modulates DNA damage response (DDR) protein stability in both cancerous and non-transformed mammary epithelial cells. However, this effect appears transient, as protein levels returned to baseline, indicating a potential adaptive cellular response.

**Figure 5:**
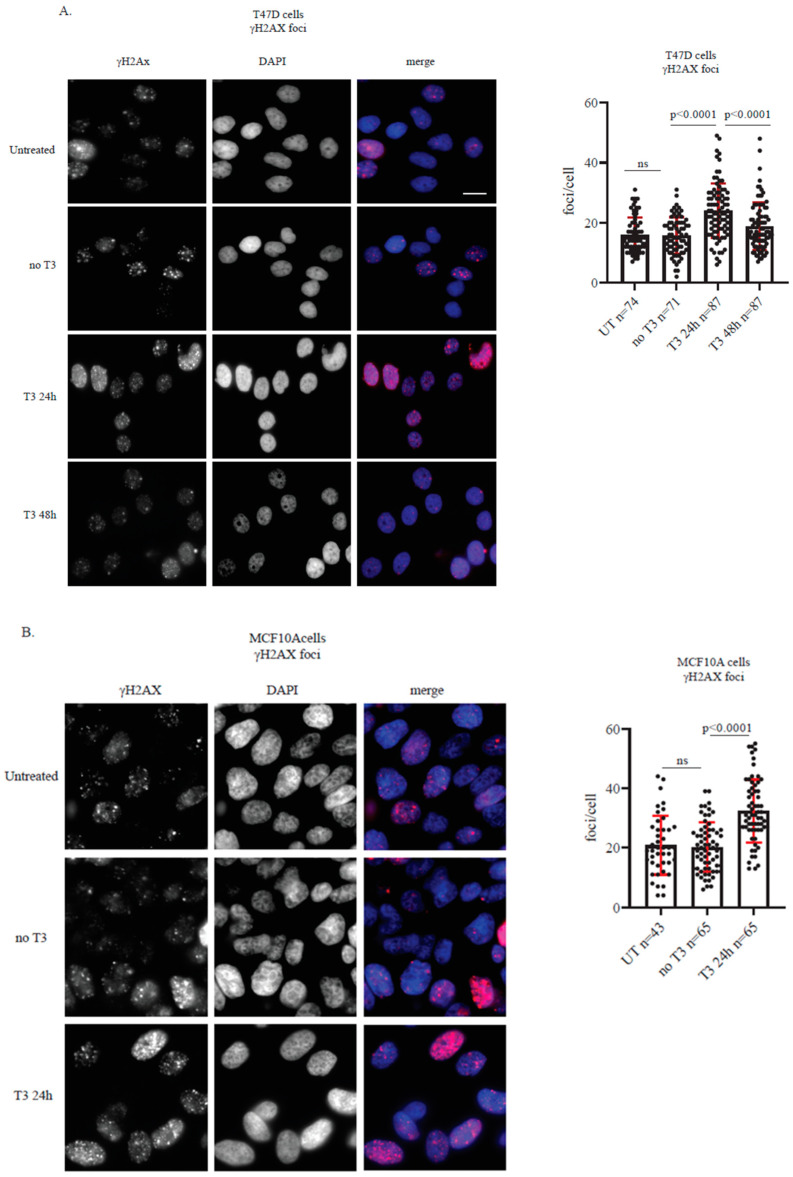
T3 Supplementation induces γH2AX foci formation. (**A**) T47D cell line presents an induction in γH2AX foci after 24 h treatment with 10 μM of T3, and a reduction after 48 h exposure. Representative images of γH2AX foci (**left panel**). γH2AX foci quantification (**right panel**). (**B**) MACF10A cell line presents an induction in γH2AX foci after 24 h treatment with 10 μM of T3. Representative images of γH2AX foci (**left panel**). γH2AX 1 foci quantification (**right panel**). For all, bar 0.5 μm, *p*-value was calculated using two-tailed *t*-test, SD = 1, ns-not significant.

To determine whether the observed upregulation of DDR proteins corresponded to an increase in DNA damage, we quantified γH2AX foci formation, a marker of DNA double-strand breaks, at 24 and 48 h with T3 treatment. Both cell lines exhibited a significant increase in γH2AX foci at 24 h, which declined to basal levels by 48 h (Figure 5). These results indicate that T3 induces transient DNA damage and/or replication stress, which is subsequently resolved or tolerated by the cells over time.

## 3. Discussion

Triiodothyronine (T3) is a central regulator of cellular metabolism, proliferation, and differentiation, exerting its effects through both nuclear receptor–mediated transcriptional programs and non-genomic signaling pathways. While thyroid hormones are generally considered essential for maintaining tissue homeostasis, accumulating evidence suggests that excessive or dysregulated thyroid hormone signaling can induce cellular stress, including oxidative stress and DNA damage [7,8,9,10]. In this study, we investigated the impact of acute T3 exposure on DNA damage response (DDR) pathways in breast cancer and non-tumorigenic mammary epithelial cells.

Our transcriptomic analysis revealed enrichment of multiple DNA repair–related pathways following short-term T3 treatment, accompanied by upregulation of RAD51, a key mediator of homologous recombination. These findings were corroborated at the protein level in both T47D and MCF10A cells, supporting activation of DDR-associated signaling. In parallel, we observed increased formation of γH2AX and 53BP1 nuclear foci, consistent with activation of DNA damage sensing and repair mechanisms. Importantly, these effects were transient, resolving within 48 h, suggesting an adaptive cellular response rather than sustained DNA damage.

The transient nature of DDR marker induction is a critical aspect of our findings. γH2AX and 53BP1 foci can arise not only from irreversible DNA double-strand breaks but also from replication stress or transcription-associated DNA lesions, particularly in proliferating cells. Thus, our data indicate that acute T3 exposure induces a state of genomic stress that activates DDR signaling, rather than permanent genomic instability. This interpretation aligns with prior reports demonstrating that thyroid hormone–induced metabolic acceleration can increase oxidative stress, which, if unresolved, may trigger DDR activation [7,11]

Notably, non-tumorigenic MCF10A cells exhibited a more dynamic DDR, with pronounced induction and rapid resolution of DDR markers, whereas breast cancer–derived T47D cells showed a more moderate but consistent response. This difference may reflect altered stress tolerance, redox homeostasis, or DNA repair capacity in cancer cells, which are often adapted to chronic metabolic and oxidative stress. Such differences may be relevant when considering how thyroid hormone signaling interacts with malignant versus non-malignant cellular contexts.

Several limitations of this study should be acknowledged. First, the concentrations of T3 used exceed physiological serum levels and should be considered pharmacological. Accordingly, the present work does not model endocrine homeostasis or long-term thyroid hormone therapy but rather examines acute cellular responses to elevated T3 exposure. Notably, similar high T3 concentrations were used in previous works to demonstrate the effect of T3 on DNA damage and oxidative stress induction in peripheral blood mononuclear cells [10] and to provoke cellular responses in HEK293 cells [12]. Moreover, the use of 10 µM T3 allowed us to probe noncanonical thyroid hormone–dependent mechanisms, rather than classical physiological signaling mediated by nuclear thyroid hormone receptors.

In addition, T3 binds strongly to serum proteins, including albumin and transthyretin (TTR), substantially reducing the free hormone fraction in culture. Consequently, higher nominal concentrations can be required to achieve effective intracellular T3 levels in vitro.

Second, while prior studies have implicated mitochondrial ROS as a mediator of T3-induced DNA damage, we did not directly measure ROS levels, oxidative DNA lesions, or mitochondrial function. Therefore, our conclusions are limited to DDR activation and do not establish direct mechanistic causality. Finally, validation of additional DDR pathway components at the protein level would further strengthen pathway-level conclusions.

Despite these limitations, our findings provide experimental support for the concept that thyroid hormone signaling can modulate genomic stress responses in breast epithelial cells. In light of clinical observations linking thyroid hormone therapy to altered genomic risk scores in ER-positive breast cancer [5], these results suggest a potential cellular framework through which thyroid hormones may influence tumor biology. However, such implications should be regarded as hypothesis-generating and warrant further investigation using physiologically relevant hormone levels, in vivo models, and patient-derived samples.

## 4. Materials and Methods

### 4.1. Cell Culture

T47D cells were cultured in RPMI-1640 media containing 10% FBS, 5 µg/mL insulin and 1% penicillin/streptomycin (All from Biological Industries Ltd. (BI), Kibbutz Beit-HaEmek, Israel) MCF10A cells were cultured in DMEM/F12 (Biological Industries Ltd. (BI), Kibbutz Beit-HaEmek, Israel) supplemented with 5% HS Biological Industries Ltd. (BI), Kibbutz Beit-HaEmek, Israel,1 µg/mL hydrocortisone (Sigma, Burlington, MA, USA), 5 µg/mL insulin (Biological Industries Ltd. (B) Kibbutz Beit-HaEmek, Israel), 20 ng/mL EGF (Sigma, Rehovot, Israel) and 100 ng/mL cholera toxin (C8052-Sigma, Gillingham, UK) and 1% penicillin/streptomycin (Biological Industries Ltd. (B) Kibbutz Beit-HaEmek, Israel). All cell lines were maintained at 37 °C in a humidified incubator with 7% CO_2_.

### 4.2. Hormone Stripped Medium

For experiments, all cells were grown in 10% fetal bovine serum charcoal stripped (capricorn-scientific FBS-CS-12A-capri) with the corresponding supplements. T3 hormone (Caymanchem 30996) was added at the indicated concentration and times.

### 4.3. RNA Sequencing Analysis

RNAseq libraries of the three samples of T47D breast cancer cell lines treated with T3 for 24 h and without treatment (control) were prepared according to the manufacturer’s protocol. RNAseq libraries were sequenced by BGI Genomics Co., Ltd., using Illumina platform. FASTQC software (version 0.11.7) [13] was used for quality control of the samples’ raw reads. Adapters were removed using TrimGalore (version 0.5.0) [14]. The raw reads were aligned against the human genome (hg38) using the alignment program HISAT2 (version 2.1.0) [15]. featureCounts software (version 1.6.3) [16] was used to measure the number of reads per gene. RNA-seq generated a median of 48 million paired-end reads per sample, of which over 91% were uniquely aligned to the GRCh38 reference genome using HISAT2.

### 4.4. Normalization and Differential Expression Analysis

In order to compare gene expression between treated and control samples, counts normalization and gene filtering to remove genes with low counts were performed using the edgeR R package (version 3.40.2) [17,18]. Data transformations for RNA-seq differential expression analysis were performed using the voom transformation [19]. Limma R package (version 3.54.1) [20] was used for the detection of differentially expressed genes. A false discovery rate (FDR) of ≤0.05 and log fold change (logFC) ≥ 1 were used as the statistical cutoff for differential expression.

### 4.5. Pathway Enrichment Analysis

Gene Set Enrichment Analysis (GSEA) was performed through the WebGestalt 2024 [21] tool against the Kyoto Encyclopedia of Genes and Genomes (KEGG) pathways database. Only pathways with FDR ≤ 0.05 are shown.

### 4.6. Statistical Analysis

Statistical analyses were performed utilizing the R 4.2.2 statistical framework [22]. The Kruskal–Wallis test was used to compare mean gene expression. Plots were generated using the ggplot2 (Version 3.4.1) [23] R package. For blots and immunofluorescence, all graphs and statistical analysis were performed using GraphPad Prism 10.5.0.

### 4.7. XTT Cell Proliferation Assay

Cells were plated on 96-well plates at 3000 cells/well. After 24 h, the medium was changed to hormone-stripped medium for 24 h (Day 0), and then T3 was added at the indicated concentration for 24 h (Day 1) or 48 h (Day 2). Proliferation was monitored using XTT cell proliferation kit (Sartorius, Göttingen, Germany), following the manufacturer’s protocol. The absorbance was read using a Tecan microplate reader at 490 nm.

### 4.8. RNA Extraction

RNA was extracted from 8 × 10^6^ cells, after 24 h of treatment with 10 μM T3. RNA was extracted using Direct-zol^TM^ RNA MiniPrep Plus (R2070, ZYMO research, Irvine, CA, USA) according to the manufacturer’s protocol.

### 4.9. Western Blot and Antibodies

For immunoblotting, cells were lysed in extraction buffer containing 50 mM Tris-HCl pH 8, 150 mM NaCl, 20 mM EGTA, 50 mM NaF and 0.1% Triton X-100, supplemented with Merck^®^ 1000× protease inhibitor (539134). Cells were lysed on ice for 30 min and cleared by centrifugation at 20,000× *g* for 30 min at 4 °C. Extracts were boiled in Laemmli buffer for 5 min. Equal amounts of protein sample (30 µg) were loaded on 10% acrylamide gels and transferred to a nitrocellulose membrane (Amershem, Piscataway, NJ, USA). RAD51 (EPR4030(3) abcam) primary antibody was used at a 1:1000 dilution. Actin (clone C4 69100, MP) was used at a 1:5000 dilution. HRP IgG light chain-specific anti-mouse secondary antibody (115-035-174; Jackson ImmunoResearch Laboratories, Inc., West Grove, PA, USA) or HRP IgG light chain-specific anti-rabbit secondary antibody (211-032-171; Jackson ImmunoResearch Laboratories, Inc.) was used at 1:10,000 dilution. Where indicated, quantitative data analysis was performed with the ImageJ/Fiji software (https://imagej.net/ij/index.html, accessed on 6 December 2025, National Institute of Health, Bethesda, MD, USA) using three repetitions, and statistical significance was calculated with the one-tailed *t*-test.

### 4.10. Immunofluorescence

Cells were grown on glass coverslips in 12-well plates with the appropriate media and fixed in 4% paraformaldehyde for 10 min at room temperature. Cells were permeabilized in 0.5% Triton X-100 in PBSx1 for 10 min at room temperature and then blocked in 5% BSA in PBS-Tween 0.1% (PBST) for 1 h at room temperature. Anti-phospho-Histone H2A.X primary antibody (Mercury; 05-636-25UG) diluted 1:400 in 5% BSA in PBST was added for 1 h at room temperature. Fluorescent dye-conjugated secondary antibody was applied for 1 h at room temperature. The coverslips were washed between each step with PBSx1. Nuclei were stained with DAPI (1:2000 dilution) at room temperature in the dark for 3 min. Coverslips were mounted on glass slides and imaged using an Olympus X81 microscope (Tokyo, Japan).

## 5. Conclusions

In summary, this study demonstrates that acute exposure to triiodothyronine (T3) induces a transient activation of DNA damage response pathways in both breast cancer and non-tumorigenic mammary epithelial cells. Through transcriptomic profiling and protein-level validation, we show that T3 exposure is associated with increased expression of key DDR markers, including RAD51, γH2AX, and 53BP1. Importantly, the resolution of these responses over time suggests an adaptive stress response rather than persistent DNA damage.

While the supraphysiological nature of T3 exposure limits direct clinical extrapolation, these findings provide mechanistic insight into how thyroid hormone signaling may influence genomic stress responses in breast epithelial cells. Future studies incorporating physiological hormone concentrations, oxidative stress measurements, and in vivo systems will be necessary to determine the relevance of these observations to thyroid hormone therapy and breast cancer progression.

These results suggest that chronic hyperthyroidism or inappropriate T3 therapy may promote genomic instability and potentially increase long-term cancer risk. These findings suggest that elevated T3 exposure can influence pathways involved in genome maintenance in vitro. However, the present study does not model chronic hyperthyroidism, long-term thyroid hormone therapy, or clinical disease progression. Accordingly, any potential relevance to cancer risk or patient management remains speculative and will require validation in appropriate *in-vivo* and epidemiological studies.

Future studies should investigate the dose-dependent effects of T3 and the contribution of antioxidant pathways to mitigate its genotoxic impact.

## Data Availability

The original contributions presented in this study are included in the article. Further inquiries can be directed to the corresponding author.

## Supplementary Materials

The following supporting information can be downloaded at https://www.mdpi.com/article/10.3390/ijms27020668/s1.
