# Supplementary figures and images for: Thyroid Hormone T3 Induces DNA Damage Response in Breast Cancer Cells
Source: Int J Mol Sci. 2026 Jan 9;27(2):668. doi: 10.3390/ijms27020668 (PMC12840950; doi:10.3390/ijms27020668)

Figure S1. uncropped gels - Figure3

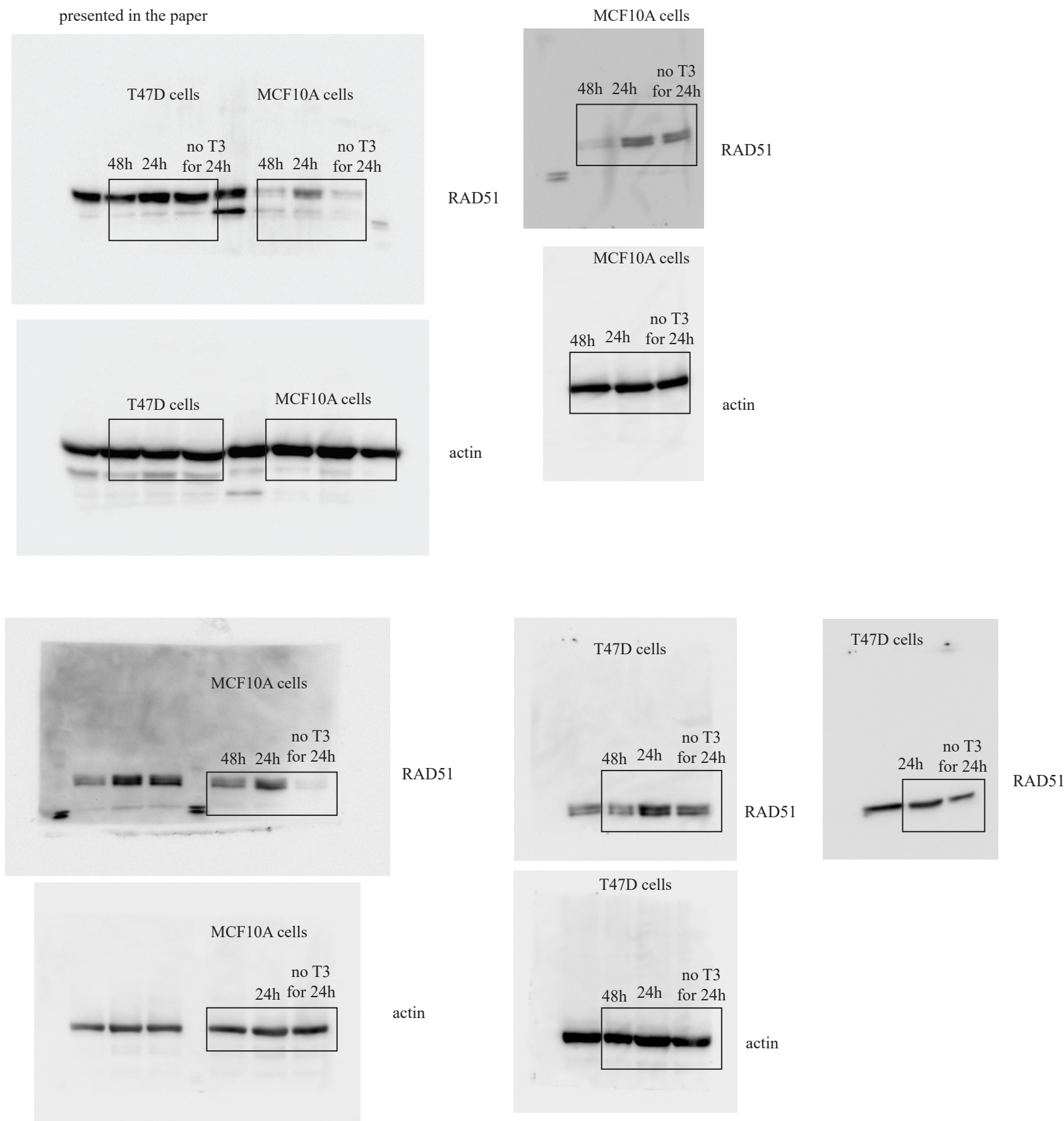

Supplement: Supplementary file 1 [file ijms-27-00668-s001.zip › ijms-4059445-supplementary.pdf]
